# Transcatheter Aortic Valve Implantation for Bicuspid Aortic Valve Disease: Procedural Planning and Clinical Outcomes

**DOI:** 10.3390/jcm12227074

**Published:** 2023-11-14

**Authors:** Lola Gutierrez, Mauro Boiago, Chiara De Biase, Omar Oliva, Pietro Laforgia, Souheib Feliachi, Alessandro Beneduce, Nicolas Dumonteil, Didier Tchetche

**Affiliations:** Groupe Cardiovasculaire Interventionnel (GCVI), Clinique Pasteur, 31300 Toulouse, France; mauro.boiago@gmail.com (M.B.); chiadebiase@gmail.com (C.D.B.); omaroliva93@gmail.com (O.O.); p.l.laforgia@gmail.com (P.L.); fsoheib@hotmail.com (S.F.); ale.beneduce@icloud.com (A.B.); ndumonteil@clinique-pasteur.com (N.D.); dtchetche@clinique-pasteur.com (D.T.)

**Keywords:** aortic stenosis, bicuspid aortic valve, transcatheter aortic valve implantation

## Abstract

Bicuspid aortic valve (BAV) is the most common congenital heart disease, with a prevalence of 1–2% and occurring in >20% of octogenarians referred for aortic valve replacement. However, BAV patients have been systematically excluded from pivotal randomized trials. Since TAVI indications are moving toward low-risk patients, an increase in the number of BAV patients who undergo TAVI is expected. BAV represents a challenge due to its unique morphological features (raphe, extreme asymmetrical valve calcifications, cusp asymmetry and aortopathy) and the lack of consensus about the accurate sizing method. The role of multi-slice computed tomography (MSCT) in the planification of the TAVI procedure is well-established, being useful to define the optimal valve sizing and the implantation strategy. New-generation devices, more experience of the operators and better planification of the procedure have been associated with similar clinical outcomes in bicuspid and tricuspid patients undergoing TAVI.

## 1. Introduction

Bicuspid aortic valve (BAV) is the most common congenital heart disease, with a prevalence of 1–2% in the general population [1]. BAV anatomy predisposes the patient to accelerated valve degeneration, which usually occurs at younger age and can be associated with multiple anatomical abnormalities, especially aortic dilatation [2]. For these reasons, surgical aortic valve replacement (SAVR) is traditionally considered the gold-standard treatment for BAV. BAV can present a challenging anatomical scenario for transcatheter aortic valve implantation (TAVI), while having a negligible impact on isolated SAVR. However, new iterations of TAVI devices, greater accuracy of imaging-based preprocedural planning and growing experience of TAVI operators have significantly improved outcomes, suggesting there is scope for similar procedural and short-term clinical outcomes after TAVI in patients with BAV as compared to patients with tricuspid aortic valve (TAV). Given the progressive change of indication for TAVI, which is extending toward younger and lower-risk patients, the number of BAV patients evaluated for a percutaneous treatment is expected to increase.

### BAV Diagnosis and Anatomical Classification

Despite transthoracic echocardiography (TTE) often being the first imaging modality when evaluating a patient for suspected BAV, it can lead to severe BAV underestimation when used solely. Its accuracy is reported to be around 87%, or even lower when the study is of limited quality or there is heavy valve calcification [3]. Multi-sliced computed tomography (MSCT), thanks to its better spatial resolution, enables the diagnosis in an additional 20% of patients [4] and thus has become the method of choice for both BAV diagnosis and classification.

**Figure 1:**
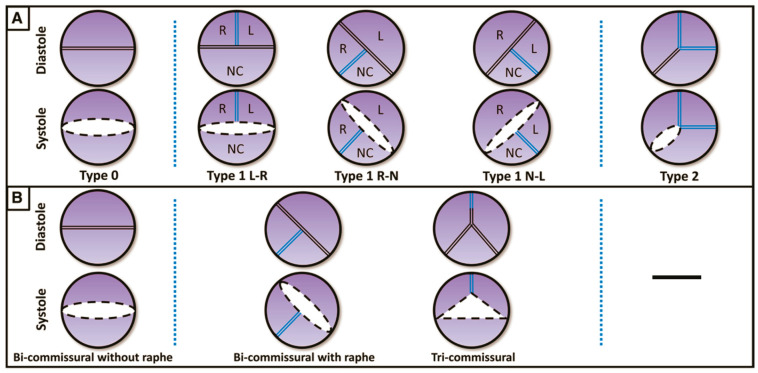
(**A**) Classification according to Sievers and Schmidtke. (**B**) Classification according to Jilaihawi. L: left coronary cusp; N: noncoronary cusp; R: right coronary cusp. (Ref [5]: A classification system for the bicuspid aortic valve).

The most widely used classification is the surgical one, described by Sievers and Schmidtke [5], which defines three types of BAVs based on the presence, number and spatial orientation of the raphe: BAV ***type 0*** (no raphe); ***type 1*** (one raphe, between two fused cusps, typically the right and left cusps), which is the most common phenotype; and ***type 2*** (two raphes) (Figure 1).

In 2016, a new classification was proposed by Jilaihawi [6] based on MSCT anatomy, describing three morphologies: ***tricommissural type***, ***bicommisural raphe type*** and ***bicommissural non-raphe type*** (Figure 1). The majority of the patients have tricommissural valves (55%), and then bicommissural with raphe is present in 41% of cases and bicommissural without raphe in the remaining 4%.

Finally, in 2021, the authors of the International Consensus Statement on Nomenclature and Classification of the Congenital Bicuspid Aortic Valve [7] suggested that BAV phenotypes include a spectrum of abnormal morphologies, which begin with the partial-fusion BAV and finish with the two-sinus BAV, determined according to the severity of the embryological mechanisms.

Regardless of the classification used, to date, no clear correlation has been demonstrated between BAV phenotypes and clinical outcomes after TAVI. A subgroup analysis from the BEAT Registry revealed a trend toward lower VARC-2 device success (72% vs. 86.7%; *p* = 0.07) and a higher rate of mean transprosthetic gradient ≥ 20 mmHg (24% vs. 6%, *p* = 0.007) in type 0 vs. type 1 BAV, potentially in relation to the more elliptical configuration of the transcatheter heart valves (THVs) [8,9].

## 2. Preprocedural Planning

MSCT plays a central role in TAVI preprocedural planning, and it is even more crucial in patients with BAV. Several studies have demonstrated that MSCT before TAVI can improve procedural results in this anatomical setting [6,10,11]. Careful evaluation of valve anatomy (annular and supra-annular dimensions, number of cusps, presence and orientation of the raphe, calcium burden and distribution, height and location of coronary ostia) and the aorta (horizontal aorta, concomitant aortic dilatation) is essential for a proper valve-type and size selection and to anticipate potentially dramatic complications.

## 3. THV Sizing

In the tricuspid anatomy, the aortic annulus is the narrowest part of the aortic root where the prosthetic valve will be sealed; hence, it is recommended that the THV should be sized based on annular dimensions, the so-called virtual basal ring (VBR). On the contrary, in a non-negligible proportion of BAV patients (approximately 13.8%), the tightest part of the aortic root may be situated above the annulus [12].

**Figure 2:**
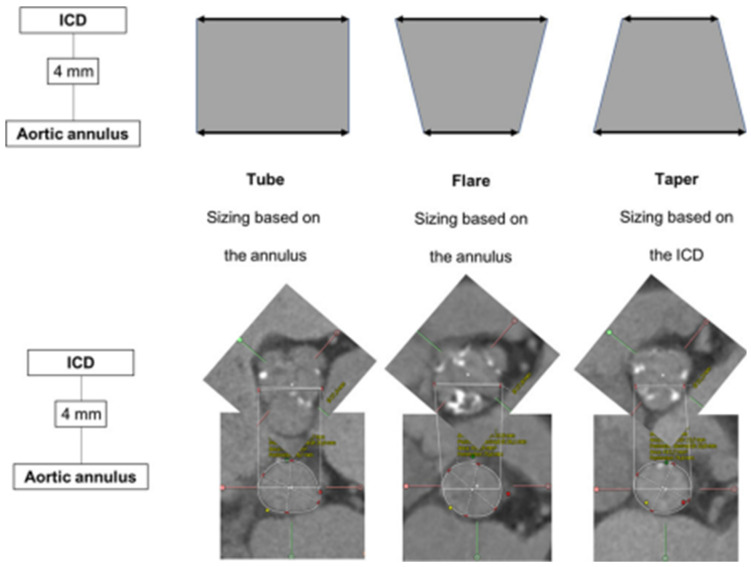
Landing zone configurations in bicuspid patients. ICD: intercommissural distance. (Ref [12]: Bicuspid Aortic Valve Anatomy and Relationship With Devices: The BAVARD Multicenter Registry). Tubular: ICD at +4 mm over the annulus is similar to the perimeter-derived diameter. Flared: ICD at +4 mm over the annulus is larger than the perimeter-derived diameter. Tapered: ICD at +4 mm over the annulus is smaller than the perimeter-derived diameter.

Tchétché et al. first developed the concept of the BAV landing zone. According to the **BAVARD** Registry [12], the evaluation of the intercommissural distance (ICD) at 4 mm above the annulus, as compared to annular dimensions, enables identifying three different landing zone configurations (flared, tubular and tapered, Figure 2). The traditional annular sizing method is appropriate in most patients with flared and tubular configurations (80–90%), but in the remaining proportion of cases with tapered configurations, the use of the supra-annular sizing method is deemed useful.

In 2020, another BAV-specific sizing strategy was proposed by Iannopollo et al. The Level of Implantation at the Raphe (**LIRA**) method [13] suggests evaluating the valve perimeter at the level of the VBR and at the level of raphe maximum protrusion, then sizing the THV according to the smallest one in case of discrepancies. This strategy was tested on 50 patients with high procedural success and good short-term clinical outcomes [14].

Petronio et al. elaborated a completely different sizing method called **CASPER** (Calcium Algorithm Sizing for bicusPid Evaluation with Raphe) [15], which focuses on three valvular features: calcium score, raphe length with respect to annular diameter and calcium localization in relation to raphe. This multiparametric algorithm was validated in a small cohort with excellent procedural results. Of note, the LIRA and CASPER methods are applicable only in raphe-type BAV.

**Figure 3:**
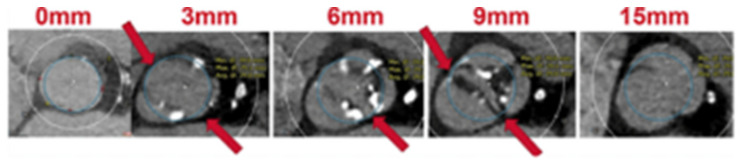
Circle method. Sapien 3 simulation and interaction with BAV anatomy. Circles drawn at the annular plane, 3/6/9 mm above the annulus [16].

Last, the **circle method** [16] is another sizing approach specific to BAV patients treated with balloon-expandable valves (BEVs). This technique consists of drawing circles, with an identical diameter to the prosthetic valve size, every 3 mm above the annular plane (Figure 3). The circles drawn permit an understanding of the interactions between the prosthesis and the anatomical structures and mean future complications can be predicted. Three options can be observed: if the circles are bigger than the valve anatomy, there is a risk of annular rupture; if the circles touch the anatomy, good sealing is expected; and if the circles are smaller than the valve anatomy, there is a risk of paravalvular leak (PVL) and THV embolization.

The concept of supra-annular sizing remains debated and poorly standardized, with significant variability across operators. Recent evidence from the prospective BIVOLUT-X registry [17], comparing annular and combined (annular + supra-annular) sizing in patients with BAV treated with SEV, suggests that combined sizing is associated with similar clinical outcomes and device success rates to those obtained with conventional annular sizing. Larger studies comparing various sizing methods are required to identify the most accurate strategy. In this regard, the results of the ongoing AD HOC retrospective registry are highly awaited.

## 4. THV Selection

The selection of the most appropriate valve for each patient should be based on device-specific features and patient-specific anatomical aspects. The Sapien platform allows very stable deployment and predictable positioning. Its great radial force usually permits circular THV frame expansion, at the cost of higher risk of annular rupture, especially in heavily calcified anatomy. The flexibility of the delivery system and the short stent frame can reduce the risk of aortic injury in the case of aortic dilatation. On the other hand, in self-expanding valves (SEVs), the possibility of repositioning and recapturing allows one to achieve an accurate positioning. The supra-annular design provides a better hemodynamic performance in constrained anatomy. Yet, the lower radial force might lead to THV underexpansion and a higher degree of PVL. To date, the BEAT Registry [18] is the only study that has attempted to directly compare the safety and efficacy of BEV (Sapien3) versus SEV (Evolut R/PRO) in the context on BAV stenosis, revealing similar rates of device success (BEV 85.7% vs. SEV 84.4%, *p* = 0.821) and clinical outcomes up to 1 year [18]. Better hemodynamic performance was observed among patients treated with SEV, but also a higher rate of significant PVL (BEV 0% vs. SEV 9.3%, *p* = 0.043). Specific data on SEV (Evolut R/PRO) have recently been published in the BIVOLUTX Registry [17], which reported an excellent device success rate (91.3%) and valve intended hemodynamic performance (95.3%), which persisted up to 1 year. A study has also demonstrated that the self-expanding Accurate platform is a feasible and safe alternative in BAV anatomy [19].

## 5. Procedural Considerations

### 5.1. Balloon Valvuloplasty

Gentle pre-dilatation with a semi-compliant balloon sized according to the smaller annular diameter is highly recommended in BAV patients, especially when using SEV or in patients with heavy valve calcifications. It can facilitate THV crossing through the stenotic native valve, offer useful information for valve sizing and positioning and allow the risk of coronary occlusion to be anticipated. In “gray zone” cases, valvuloplasty with simultaneous aortography could be used to confirm the most appropriate valve size and identify the best implantation depth according to the sealing point (waist of the balloon). Oversizing must be avoided, especially in severe calcified anatomies, to prevent intraprocedural complications.

### 5.2. Implantation Technique

Two different landing zones can be identified according to the three possible BAV configuration patterns. In tubular and flared configurations, the annulus plane is the optimal landing zone, while in the tapered pattern, a supra-annular landing zone is recommended. Higher implants have been associated with lower rates of permanent pacemaker implantation (PPI) (reducing the interaction of the THV frame with the conduction system), but at the cost of increased risk of THV embolization and coronary obstruction [12,16,20]. On this matter, despite being generally associated with high coronary ostia and large sinuses of Valsalva, in BAV patients, the presence of long and heavily calcified leaflets, bulky calcium deposits and eccentric origin of coronary ostia are potential risk factors for coronary obstruction.

Considering the younger age of BAV patients, when using SEV platforms, commissural alignment is strongly recommended as it can facilitate coronary reaccess for future revascularization, even in the case of TAVI-in-TAVI.

**Figure 4:**
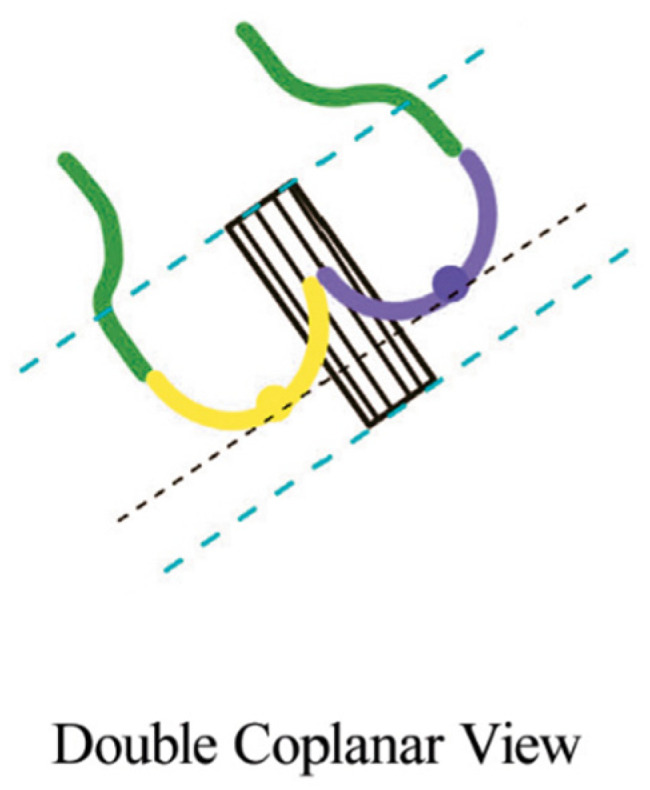
Double coplanar view. Alignment of the nadirs of the 2 cusps (black lines). The parallax at the inflow and outflow levels should be removed (blue lines) [20].

While cusp-overlap projection is the preferred deployment view for SEV platforms such as Evolut R/PRO and Navitor, the coplanar (or three-cusp) view is the gold-standard deployment projection for BEV. The alignment of the cusp in BAV anatomies can be challenging in case of severe cusp asymmetry, especially in type 0 BAV. In this case, the coplanar view is not feasible and “*double coplanar view*” alignment (both the cusp and the THV) represents the optimal deployment projection [20]. To achieve that, once the nadirs of the cusps are aligned, the stent of the THV should also be aligned without the parallax of the TVH frame (Figure 4).

### 5.3. THV Positioning

**Figure 5:**
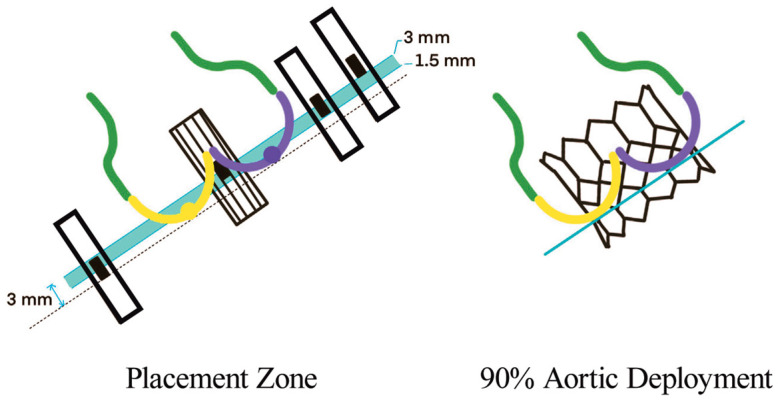
BEV deployment in BAV, where 90/10 aorta/ventricle can be achieved by positioning the radio-opaque marker slightly above the annulus [20].

In BAV patients, a higher implantation depth should be attempted, while balancing the risk of THV malpositioning and embolization [21]. It is suggested that SEV should be placed at 0–2 mm below the annulus and BEV at 90:10 (stent frame height 90% aortic, 10% ventricular). This strategy reduces the risk of THV migration toward the left ventricle and conduction disturbances. To achieve this position with BEV, the center marker (the 3 mm radio-opaque marker placed in the middle of the deployment balloon) should be placed slightly above the line between the two or three bases of the cusps (Figure 5). However, the final implantation height depends on the foreshortening of the THV frame (5.5 mm for 20 mm, 6.5 mm for 23 mm, 7 mm for 26 mm and 8.5 mm for 29 mm Sapien THV).

### 5.4. Balloon Postdilatation

**Figure 6:**
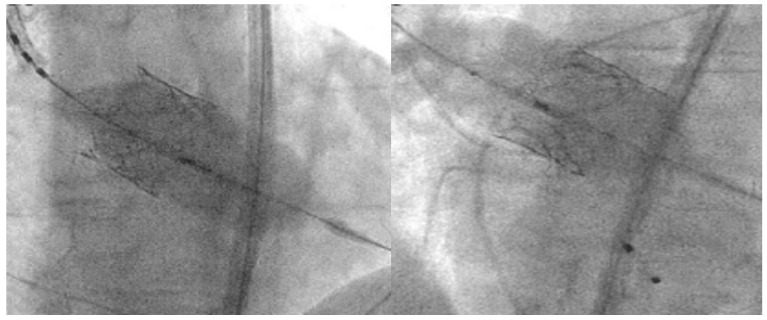
Left: “**Flare the inflow**” technique. Position the balloon toward the inflow with the radio-opaque marker at the ventricular end of the THV. Right: “**Flare the outflow**” technique. Position the balloon toward the outflow with the middle marker at the aortic end of the THV.

THV underexpansion with a residual high gradient and significant PVL can be quite a common complication in BAV patients, especially when using SEV [20,22]. This problem can usually be addressed with cautious balloon postdilatation, while aggressive postdilatation should be avoided as it exposes the patient to a higher risk of annular rupture, coronary obstruction and damage to the THV leaflets. On this matter, postdilatation with the “*flare the inflow*” technique [20] can be very useful as it enhances the inflow part of the THV, maintaining circularity without applying pressure on the annulus (Figure 6).

### 5.5. Large Annuli

In BAV anatomy, the prevalence of large annuli outside the range of currently available THVs is not rare. In these cases, overexpanding and overfilling remains a safe alternative [16,20]. The THV structure allows for overexpansion without increasing the risk of central aortic regurgitation (2–4 mL + nominal volume − 33 mL − in 29 mm Sapien 3 allows large annuli of 31 mm mean diameter to be treated). However, large annuli increase the risk of THV malposition and embolization. In these cases, postdilatation following “*the flare outflow*” technique [20] allows for achieving proper sealing and anchoring of the THV (Figure 6).

When applying the previous concepts, in “gray zone” annular sizing, a smaller or bigger THV could be chosen. In the first case, deployment with intentional overexpansion could be put into practice. Postdilatation following the “flare the inflow” technique with additional filling allows for achieving overexpansion (and reduces the risk of PVL) without increasing the pressure on the annulus. If a bigger THV is chosen, the deployment should be carried out with intentional underexpansion. Intentional underfilling of the deployment balloon is required, followed by postdilatation using the “flare the inflow” technique with a nominal volume.

## 6. Clinical Outcomes after TAVI in BAV Patients

SAVR is considered the gold-standard treatment for BAV stenosis [23,24]. BAV is frequently associated with concomitant aortopathy and tends to occur in younger and lower-risk patients, who are optimal candidates for a surgery. Furthermore, BAV might represent a complex anatomical scenario with potential negative short- and long-term implications for TAVI, while having negligible impact on isolated SAVR. Hence, BAV patients have been excluded from pivotal randomized trials comparing TAVI and SAVR. The best evidence can be derived from two propensity-matched analyses [25,26], which reported similar rates of mortality and stroke up to 1 year, higher risk of acute kidney injury and new-onset atrial fibrillation after SAVR, but more PPI after TAVI. According to a recent consensus document [21], TAVI can be a reasonable option to treat patients aged >70 years without significant aortopathy.

Comparisons between outcomes deriving from TAVI in BAV and TAV patients are limited to retrospective registries’ data, and the highest level of evidence is limited to propensity-matched analyses and meta-analyses.

In an early TAVI experience of using first-generation THV in high-risk patients, the authors reported similar mortality to TAV patients, but a lower rate of device success, greater need for conversion to surgery and higher incidence of significant PVL [10]. Since then, the introduction of new-generation THV, growing operator experience and the widespread use of imaging-based preprocedural planning have significantly improved outcomes after TAVI. Recently, two propensity-matched analyses have compared outcomes after TAVI in BAV and TAV patients at intermediate surgical risk included on the TVT Registry and treated with BEV (Sapien 3) [27] or SEV (Evolut R/PRO) [28]. Similar 1-year mortality was observed (BEV 10.5% vs. 12.0%, 95%CI 0.73–1.10; SEV 10.4% vs. 12.1%, *p* = 0.63), but a higher rate of conversion to surgery occurred in BAV patients treated with BEV, and there was a greater incidence of significant PVL and need for reintervention in BAV patients treated with SEV. The latest evidence has demonstrated favorable clinical outcomes after TAVI in selected low-risk patients. A propensity-matched analysis including patients from the Evolut Low Risk Bicuspid study revealed no difference in all-cause mortality, stroke and echocardiographic results at 1-year of follow-up [29]. An equivalent propensity-matched analysis was conducted with patients included in the PARTNER 3 Bicuspid Registry, showing similar rates of the composite endpoint of death, stroke and cardiovascular rehospitalization at 1 year (10.9% versus 10.2%; *p* = 0.80) [30].

Ultimately, the outcomes after TAVI in BAV patients appear to be strongly dependent on specific anatomical features. Thus, appropriate patient selection is crucial to obtain satisfactory results, as underscored by a recent meta-analysis from Montalto [31].

Among these features, the calcification amount and distribution should be important considerations before valve treatment. Severe calcifications are commonly observed in BAV anatomy and both the amount and, above all, distribution of calcium can predict procedural complications. In particular, the presence of a calcified raphe can affect prosthesis expansion and can increases the risk of PVL and THV malpositioning. Heavy calcifications in the commissural zones entail higher risk of annular rupture. Finally, bulky leaflet calcifications can be associated with stroke and coronary obstruction. This is even though BAV patients usually have wide sinuses of Valsalva, which protect them from acute coronary occlusion during TAVI.

**Figure 7:**
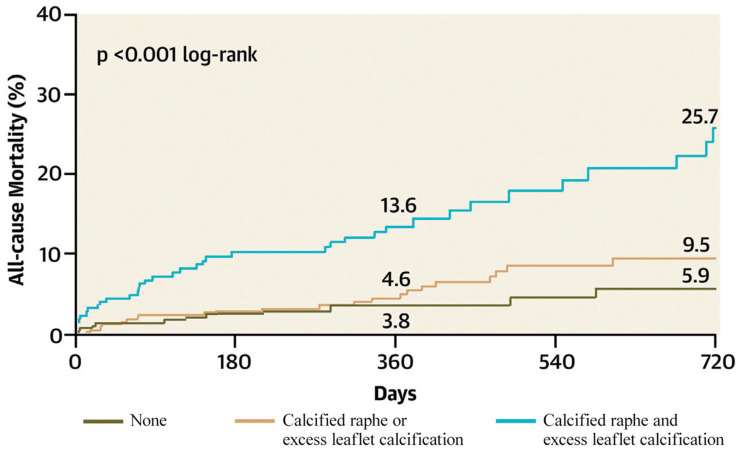
All-cause mortality according to the BAV features (Ref. [22], Bicuspid Aortic Valve Morphology and Outcomes After Transcatheter Aortic Valve Replacement. Yoon, S.-H et al.).

Yoon et al. identified a calcified raphe and excess leaflet calcification as independent predictors of procedural complications and 2-year all-cause mortality. Patients with both morphological characteristics had higher rates of aortic root injury, moderate-to-severe PVL and 30-day mortality [22] (Figure 7).

Significant PVL was originally a major limitation of TAVI, especially in BAV patients, who showed an even higher rate of PVL when first-generation devices were used (BAV 15.9% versus TAV 10.3, *p* = 0.003) [32]. However, the improvement of valve sealing performance in new-generation devices has drastically reduced the incidence of this problem (BAV 2.7%).

Conversely, the rate of PPI has not significantly decreased, despite the introduction of new-generation devices. This complication is traditionally higher when using SEV as compared to BEV. Despite the Bivolut X Registry and TAVR Low Risk Bicuspid Study [33] reported significantly higher rates of PPI in BAV patients treated with SEV as compared to TAV patients, according to a recent metanalysis [34], the incidence seems to be independent of valve morphology (RR 1.06, 95%CI 0.94–1.21). New evidence produced by using the cusp overlap technique for SEV implantation in BAV patients could be of interest [35,36].

The embolization of small debris during valve implantation can lead to periprocedural stroke. High valve calcification and additional procedural manipulation (pre-/postdilatation) can further increase the risk. According to the TVT Registry, the incidence of cerebral ischemic events was not significantly different among BAV and TAV patients treated with last-generation BEV [27]; however, an increased risk has been described in another series [31].

After the publication of the results of the PROTECTED TAVR trial [37], the role of the cerebral embolic protection device (CEPD) remains uncertain. The use of the CEPD during TAVI did not translate into significant differences in the incidence of periprocedural stroke or TIA. However, considering the potential disabling effect of this complication, a systematic use of CEPD may be considered in young BAV patients.

THV underexpansion in hostile BAV anatomy can affect THV durability and increase the risk of leaflet thrombosis due to accelerated prosthetic degeneration, a matter of particular concern in younger BAV patients. Long-term data beyond 1 year of follow-up are still scarce and highly awaited.

## 7. Conclusions

Since TAVI indications are moving toward young and low-risk patients, the number of BAV patients evaluated for percutaneous treatment is expected to increase.

Accurate imaging-based preprocedural planning has been proved to enhance outcomes after TAVI. It appears even more crucial in BAV anatomy to identify the appropriate valve size and the intended implantation level according to the landing zone configuration. This also enables identifying unfavorable anatomical features that can predict potentially dramatic complications and poor outcomes.

Experienced TAVI operators can now offer similar procedural and short-term clinical outcomes to selected BAV patients as compared to TAV patients. However, favorable results after TAVI remain strictly dependent on careful patient selection. Long-term evidence is highly awaited.
